# Ultrastructure of Exospore Formation in *Streptomyces* Revealed by Cryo-Electron Tomography

**DOI:** 10.3389/fmicb.2020.581135

**Published:** 2020-09-24

**Authors:** Danielle L. Sexton, Elitza I. Tocheva

**Affiliations:** Department of Microbiology & Immunology, Life Sciences Institute, The University of British Columbia, Vancouver, BC, Canada

**Keywords:** microbial ultrastructure, cryo-electron tomography, *Streptomyces*, multicellular bacteria, filamentous bacteria, sporulation, cell envelope, bacterial cytoskeleton

## Abstract

Many bacteria form spores in response to adverse environmental conditions. Several sporulation pathways have evolved independently and occur through distinctive mechanisms. Here, using cryo-electron tomography (cryo-ET), we examine all stages of growth and exospore formation in the model organism *Streptomyces albus*. Our data reveal the native ultrastructure of vegetative hyphae, including the likely structures of the polarisome and cytoskeletal filaments. In addition, we observed septal junctions in vegetative septa, predicted to be involved in protein and DNA translocation between neighboring cells. During sporulation, the cell envelope undergoes dramatic remodeling, including the formation of a spore wall and two protective proteinaceous layers. Mature spores reveal the presence of a continuous spore coat and an irregular rodlet sheet. Together, these results provide an unprecedented examination of the ultrastructure in *Streptomyces* and further our understanding of the structural complexity of exospore formation.

## Introduction

**FIGURE 1:**
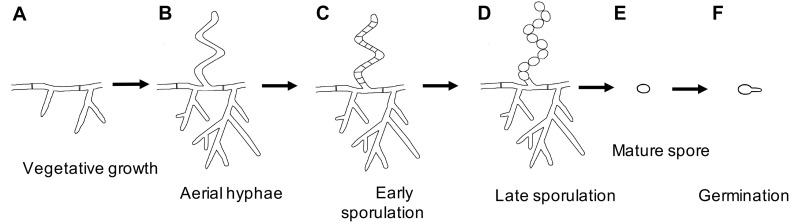
*Streptomyces* life cycle. Schematic representation of the major growth stages: **(A)** Vegetative hyphae, **(B)** Early sporulation begins with aerial hyphae formation, **(C)** Synchronous septa formation to produce immature spores, **(D)** Spore maturation, **(E)** Release of mature spores, and **(F)** Germination into vegetative hyphae.

Bacterial sporulation encompasses a diverse set of developmental processes which culminate in the production of specialized dormant life forms called spores. Spores are morphologically distinct from vegetative cells, often having additional protective structures on the surface such as modified peptidoglycan (PG) and proteinaceous layers. Several independent mechanisms for bacterial sporulation have evolved. The most extensively characterized mode of sporulation, both genetically and structurally, is endospore formation in Firmicutes, exemplified by *Bacillus subtilis* (Higgins and Dworkin, 2012; Tocheva et al., 2013; Khanna et al., 2019). Exospore formation, on the other hand, has been extensively characterized in the multicellular bacteria *Streptomyces*, members of the phylum Actinobacteria. *Streptomyces* grow vegetatively as a series of interconnected multinucleate compartments, forming multicellular branching filamentous hyphae. Nutrient limitation triggers sporulation and the process begins by the emergence of specialized non-branching aerial hyphae from the colony surface (McCormick and Flardh, 2012; Figure 1). The hyphae undergo synchronous cell division to produce numerous identical spores. Mature spores are released into the environment to ensure dispersal of genomic material. While *Streptomyces* sporulation is phenotypically similar to many filamentous fungi, these processes are the result of convergent evolution.

In the soil, streptomycetes exist predominantly as spores that remain dormant until favorable growth conditions are sensed (Ensign, 1978). The exact nutrient set required for germination remains unknown, however, some studies have shown that divalent cations such as Ca^2+^ and Mg^2+^ can induce germination (Ensign, 1978; Eaton and Ensign, 1980). Following the cue to initiate germination, spores rehydrate and switch from phase-bright to phase-dark when viewed with phase-contrast light microscopy. This stage proceeds without new cell wall synthesis (Ensign, 1978). When a new vegetative cell emerges from the spore in the form of a germ tube (Figure 1F), degradation of the spore wall and synthesis of new PG at the tip is required (Flardh, 2003; Sexton et al., 2020). It has been shown previously that polar growth of *Streptomyces* is directed by the polarisome, a complex containing DivIVA, Scy, and FilP (Flardh, 2003; Fuchino et al., 2013; Holmes et al., 2013; Figure 1A). DivIVA is a coiled coil protein that forms filaments *in vitro* and localizes to sites of negative curvature *in vivo* (Hempel et al., 2008; Lenarcic et al., 2009). In *B. subtilis*, it has further been shown that DivIVA interacts directly with the inner leaflet of the cytoplasmic membrane (Stahlberg et al., 2004; Oliva et al., 2010). Scy is another coiled coil protein that forms filaments, and its interaction with DivIVA is thought to control the frequency of branching during vegetative growth (Holmes et al., 2013) and its interaction with ParA is thought to anchor a copy of the chromosome to the extending hyphal tip (Ditkowski et al., 2013; Kois-Ostrowska et al., 2016). The last member of the polarisome, FilP, is shown to form intermediate filaments and aid in several important molecular processes, including polarisome stabilization (Frojd and Flardh, 2019) and hyphal tip rigidity (Bagchi et al., 2008; Fuchino et al., 2013). Furthermore, the polarisome likely guides the localization of penicillin binding proteins and the glycosyltransferase CslA involved in the synthesis of new PG and cellulose-like polymer, respectively (Xu et al., 2008; Holmes et al., 2013; Ultee et al., 2020). Overall, the polarisome is a key protein complex that coordinates molecular processes important for polar growth.

Filamentous bacteria, such as cyanobacteria, have evolved septal junctions that facilitate communication between neighboring cells (Merino-Puerto et al., 2011; Wilk et al., 2011). Similarly, in *Streptomyces*, vegetative hyphae are occasionally subdivided into linked syncytial compartments by vegetative septa (McCormick and Flardh, 2012). Plasmid DNA and GFP have been shown to traverse vegetative septa (Kataoka et al., 1991; Hopwood and Kieser, 1993; Celler et al., 2016), suggesting that vegetative septa are selectively permeable to these molecules or that proteins and DNA are actively transported across vegetative septa. While vegetative septa are not required for growth or viability (McCormick et al., 1994), they may confer other advantages. For example, as the colony prepares for sporulation, vegetative hyphae in the center of the colony undergo lysis (Miguelez et al., 1999). This is thought to provide valuable nutrients that fuel sporulation. Compartmentalizing the vegetative hyphae with selectively permeable septa could allow for secure transport of valuable nutrients through the cytoplasm of the hyphal network to sites of sporulation. Notably, the presence of septal junctions in *Streptomyces* vegetative septa that could allow for transport of molecules has been suggested but not confirmed (Strunk, 1978; Jakimowicz and van Wezel, 2012).

The first step in sporulation is the production of aerial hyphae above the colony surface (Figure 1B). In order to lower the surface tension at the air-colony interface, the aerial hyphae are coated with a hydrophobic layer composed of rodlin and chaplin proteins (Claessen et al., 2003, 2004; Elliot et al., 2003). Without this rodlet layer, colonies are unable to produce aerial hyphae or sporulate (Claessen et al., 2003; Elliot et al., 2003). Once aerial hyphae cease lengthening, they are subdivided by septa to produce immature spores (Figure 1C). Septal formation is directed by FtsZ, which synchronously forms numerous Z rings 1–2 μm apart inside the aerial hyphae (Schwedock et al., 1997). MreB localizes to the formed septa, possibly to aid in PG synthesis at the newly formed septa (Mazza et al., 2006). Subsequently, MreB and other components of the *Streptomyces* spore-wall synthesizing complex (SSSC) localize around the entire spore to direct spore wall synthesis (Figure 1D; Mazza et al., 2006; Kleinschnitz et al., 2011). It is yet unclear how much of the existing PG is modified to become a part of the spore wall. Once the maturation process is complete, spores are released from the spore chain, likely by mechanical forces (Figure 1E).

Previous characterization of *Streptomyces* development has been done using traditional electron microscopy (EM) techniques (Glauert and Hopwood, 1960, 1961; Hopwood and Glauert, 1960; Bradley and Ritzi, 1968; Wildermuth and Hopwood, 1970; Wildermuth et al., 1971; McVittie, 1974; Hardisson and Manzanal, 1976; Manzanal and Hardisson, 1978; Strunk, 1978). These techniques rely on dehydration and crosslinking by fixation, which can disrupt cellular ultrastructure. With this study, we characterized all stages of *Streptomyces* growth and development using cryo-electron tomography (cryo-ET). Cryo-ET preserves cells in their native state and provides three dimensional reconstructions of whole cells at ∼4 nm resolution (Tocheva et al., 2010). By cryogenically preserving *S. albus* at different stages of their life cycle, we aimed to characterize the ultrastructure associated with vegetative and sporulating cells. Our observations include the structure of the polarisome in vegetative hyphae, septal junctions between vegetative cells, the rodlet layer on the surface of aerial hyphae and spore chains, and the presence of a spore coat surrounding mature spores. Therefore, cryo-ET provides a unique opportunity to directly observe notable cellular structures related to growth and multicellular development of *Streptomyces*.

## Materials and Methods

### Strains and Growth Conditions

*Streptomyces albus* was grown on solid soy flour mannitol agar (20 g/L soy flour, 20 g/L mannitol, and 20 g/L agar) or in liquid 2 × YT medium (16 g/L tryptone, 10 g/L yeast extract, and 5 g/L sodium chloride) supplemented with 20 mM MgCl_2_ (Kieser et al., 2000).

### Sample Preparation

For vegetative and sporulation samples, cells were grown on soy flour mannitol agar until the desired growth stage was reached. For germination, spores were resuspended in liquid 2 × YT medium supplemented with 20 mM MgCl_2_. Spores were heat shocked at 50°C for 10 min and then incubated at 30°C for 4 h, until germ tubes were observed with light microscopy. Prior to imaging, single colonies were picked off the plate, and resuspended in phosphate buffered saline pH 7.2. Images were collected using phase contrast microscopy on an upright Ziess Axio Examiner Z1 equipped with an Axiocam 506 mono camera and a 100× lens and processed using Zen Blue 2.1. To measure cell compartment lengths, membranes were stained with 1/1,000 dilution of CellBrite Fix 640 (Biotium). Cells were measured using Fiji software (Schindelin et al., 2012).

### Cryo-ET Data Collection and Processing

Samples were mixed with 20-nm colloidal gold particles, loaded onto glow-discharged carbon grids (R2/2, Quantifoil) and plunge-frozen into liquid ethane-propane mix cooled at liquid nitrogen temperatures with a Mark IV Vitrobot maintained at room temperature and 70% humidity. Tilt series of samples at all growth stages were collected using SerialEM (Mastronarde, 2005) on a Titan Krios 300 keV transmission electron microscope (Thermo Fischer Scientific) equipped with a Falcon III camera, Gatan K3 camera, or a Gatan K3 camera and Bioquantum energy filter. Tilt series were collected at 10 μm defocus, 120 e^–^/Å^2^ total dose, ±60° tilt, and 1° increments. Three dimensional reconstructions were calculated using the IMOD package and the back-weighted projection method (Kremer et al., 1996).

## Results and Discussion

**FIGURE 2:**
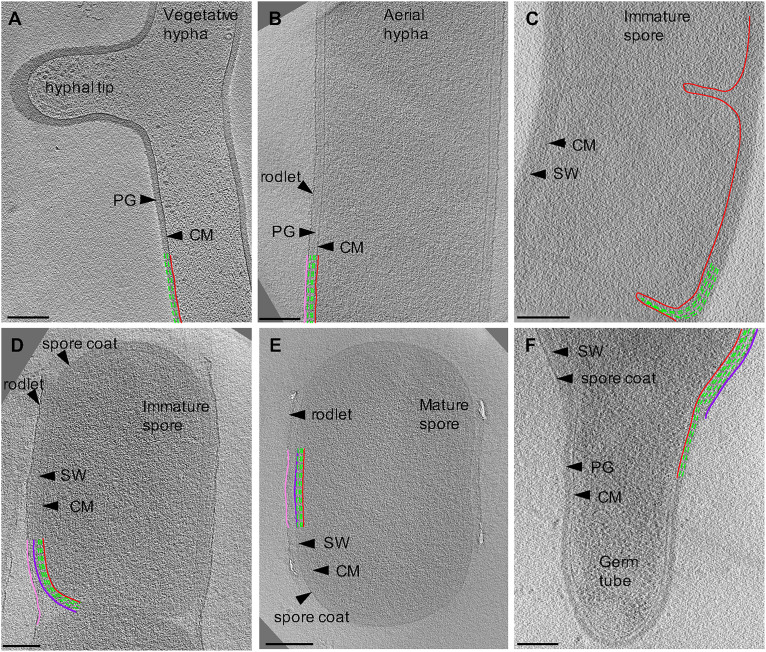
Cryo-ET of vegetative, sporulating, and germinating *Streptomyces*. Tomographic slices through *S*. *albus* at different growth stages corresponding to **(A)** vegetative hyphae, **(B)** aerial hyphae formation during early sporulation, **(C)** septa formation during early sporulation, **(D)** spore maturation, **(E)** release of mature spores, and **(F)** germination. Cytoplasmic membrane (CM) is shown in red. Vegetative peptidoglycan (PG) and spore wall (SW) are shown in green. Rodlet ultrastructure and spore coat are shown in pink and dark purple, respectively. Each tomographic slice is 20-nm thick. Scale bar = 200 nm.

To characterize exospore formation in Actinobacteria, we collected tomograms from each stage of *Streptomyces* growth (Figure 2). Model *Streptomyces* species such as *S. coelicolor* and *S. venezuelae* produce cells that are too thick (∼ 1 μm) for direct imaging with cryo-ET, so we used *S. albus* as a model system. *S. albus* vegetative hyphae are <0.5 μm in diameter and thus suitable for analysis by cryo-ET without additional thinning of the sample.

### Features of Vegetative Hyphae

First, we sought to characterize vegetative growth, and subsequently identify major cellular changes induced by sporulation. Vegetative hyphae were ∼ 0.5 μm in diameter and ran up to tens of microns in length, with occasional cross walls and membranes separating the hyphae into 10 μm long multinucleate compartments (Table 1). The vegetative PG was ∼35 nm thick (Table 1) with an exterior surface that appeared smooth and undecorated (Figure 2A). At hyphal tips, the PG was 5–20 nm thicker than the lateral region of the same tip presumably due to deposition of additional layers of polysaccharides and teichoic acids (Figure 2A, Table 1, and Supplementary Movie S1) (Ultee et al., 2020). Other than the variation of thickness at the tip, the cell wall appeared uniform (Figure 2A). Our results were consistent with reports on *S. coelicolor* sacculi, where the apical region appeared thicker than the lateral wall (Ultee et al., 2020), however, we did not observe distinct lamellae of cellulose-like polymers and PG at the hyphal tips in *S. albus.* This difference in observations could be due to the experimental conditions used by the two studies. While both studies used cryo-ET, we visualized whole cells under turgor pressure whereas Ultee et al. (2020) imaged thin purified sacculi that were boiled in SDS prior to imaging.

**TABLE 1:** Cell dimensions, PG thickness, and septum thickness at each developmental stage.

Growth stage	Cell width (nm)^1^	Cell length (μm)^1^	PG thickness (nm)^1^	Septum thickness (nm)^1^	PG thickness at tip (nm)^1^	Number of tomograms
Vegetative hyphae	515 ± 75	10 ± 4	35 ± 5	40 ± 4	40 ± 14	20
Aerial hyphae	815 ± 5	n/a	50 ± 5	n/a	n/d	16
Early sporulation (pre division)	995 ± 5	0.9 ± 0.2	n/d^2^	48 ± 7	n/d	2
Immature spores (post division)	825 ± 20	0.9 ± 0.2	50 ± 10	n/a	n/a	4
Mature spores	795 ± 95	0.9 ± 0.2	60 ± 10	n/a	n/a	14
Germination	n/a^3^	n/a	62 ± 4 for spore/35 ± 5 for germ tube	n/a	n/d	6

*^1^Values are average ± one standard deviation. ^2^n/d, not determined. ^3^n/a, not applicable.*

**FIGURE 3:**
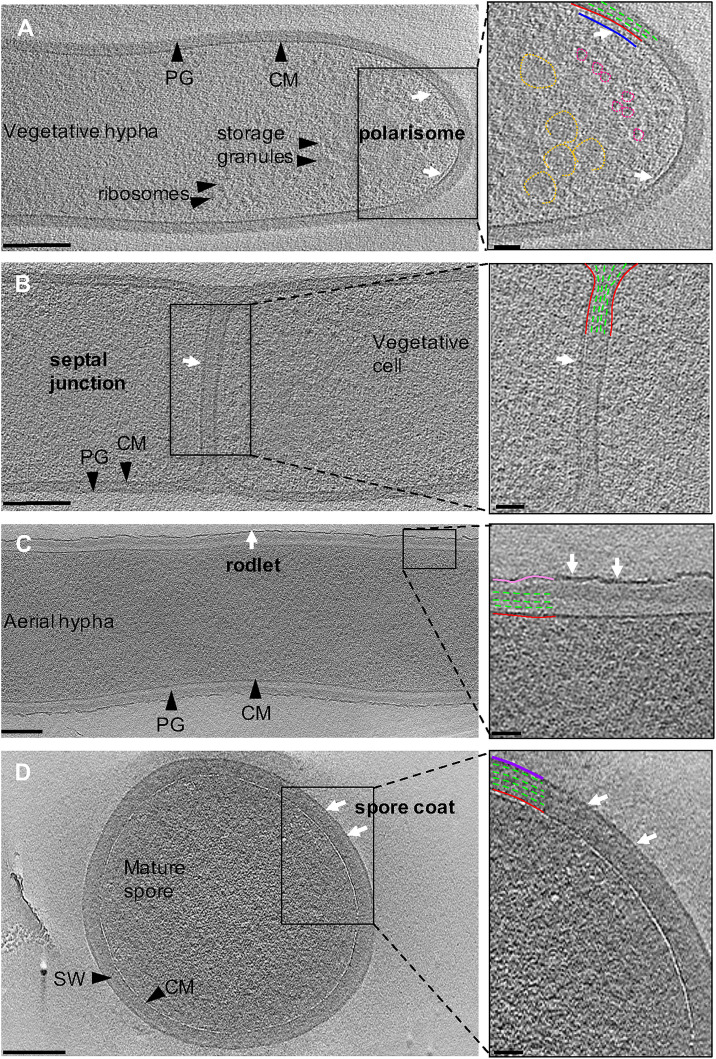
Ultrastructure of *Streptomyces*. Tomographic slices through *S. albus* cells at different stages of sporulation. **(A)** Vegetative hypha. The cytoplasmic membrane and peptidoglycan are shown in red and green, respectively. Putative features include: polarisome (blue, white arrows), glycogen storage granules (yellow), and ribosomes (pink). **(B)** Vegetative septum between two neighboring cells. A putative septal junction is highlighted with white arrows. **(C)** Aerial hypha reveal the expected rodlet layer on the surface (pink, white arrows). **(D)** Mature spore surrounded by a 10-nm thick layer, supposed to be a spore coat (purple, white arrows) on the surface of the spore wall (SW). Insets show a magnified image of the boxed areas. Scale bar = 200 nm, inset 50 nm.

Ribosomes and storage granules were observed throughout vegetative hyphae (Figure 3A). The storage granules in *S. albus* were likely composed of glycogen as glycogen has been shown to accumulate during vegetative growth to fuel sporulation in other streptomycetes (Brana et al., 1986; Rueda et al., 2001). We observed a 6-nm wide layer ∼10 nm underneath the cytoplasmic membrane at hyphal tips that was not detected elsewhere in the vegetative hyphae (Figure 3A, inset and Supplementary Movie S2). We predict that the layer represents a putative polarisome and thus, the observed structure likely represents a complex of DivIVA and Scy (Holmes et al., 2013). While purified FilP formed striated bundles *in vitro* when imaged with negative staining EM (Soderholm et al., 2018; Javadi et al., 2019), we did not observe such structures in our tomograms, suggesting that FilP may adopt alternate conformations under native conditions or in association with other proteins. Additional filaments were occasionally observed in the cytoplasm, along the length of the vegetative hyphae and near hyphal tips (Supplementary Figure S1 and Supplementary Movie S2). Due to the orientation of these filaments (along the cell length) and their distance away from the membrane (∼80 nm), it is unlikely that they are composed of MreB or FtsZ. Even though the nature of these filaments is unknown, they suggest that a cytoskeletal protein is expressed and involved in a cellular process during vegetative growth at tip of hyphae. Collectively, our observations highlight the complex interplay of cytoskeletal proteins at the hyphal tip to co-ordinate growth and chromosome positioning inside the cell.

*Streptomyces* hyphae are subdivided into compartments by occasional septa. Our tomograms showed that vegetative septa divided hyphae into compartments without completing cell-cell separation (Figure 3B). PG in septa was continuous with the vegetative cell wall and appeared visually similar, suggesting comparable composition and structure of the two. The septa appeared slightly thicker (∼50 nm) than the surrounding cell wall of ∼35 nm (Table 1), which could be due to the difference in the macromolecular machinery guiding the processes (McCormick et al., 1994; Mistry et al., 2008). Several cryotomograms of vegetative septa revealed 12-nm wide septal junctions with a 9-nm wide lumen (Figure 3B inset, Supplementary Figures S2, S3, and Supplementary Movie S3), which could allow free flow of small molecules, proteins, and DNA between adjacent cells. The septal junctions we identified in our tomograms appeared similar to the suggested septal junctions in *S. melanochromogenes* (Strunk, 1978). Of the 8 septa we examined, four had 2–5 septal junctions per septum and four did not appear to have any. These observations align with previous studies where GFP was able to traverse some but not all vegetative septa in *S. coelicolor* (Celler et al., 2016). Structures of similar septal junctions composed of Fra family proteins and SepJ were recently reported in filamentous cyanobacteria (Weiss et al., 2019). Homologs of these proteins do not exist in *S. albus*, suggestive of a different mechanism for cell-to-cell communication in *Streptomyces*.

### The Cell Envelope Undergoes Dramatic Remodeling During Sporulation

Sporulation in *Streptomyces* is triggered by nutrient limitation (McCormick and Flardh, 2012). The first step of the process involves the formation of morphologically distinct aerial hyphae. The aerial hyphae of *S. albus* were on average 300-nm thicker than the vegetative cells (Table 1). As a result, less detail was resolved in the cytoplasm of these cells. Some of the notable changes were observed in the cell envelope morphology and composition. The aerial hyphae had thicker PG compared to vegetative cells (Figures 2B,C and Table 1). In addition, the rodlet layer that is integral to the formation of sporulating cells (Claessen et al., 2002, 2003; Elliot et al., 2003) was observed on the cell surface of aerial hyphae (Figure 3C). During aerial hyphae growth, the rodlet layer appeared discontinuous (Figure 3C, inset), reflective of the overlapping basket weave structure previously reported on the surface of aerial hyphae and mature spores (Claessen et al., 2003; Elliot et al., 2003).

Cryotomograms of aerial hyphae showed synchronous formation of sporulation septa during early sporulation and the development of immature spores (Figure 2C). At this stage, the cells were ∼1 μm in diameter, ∼200-nm thicker than aerial hyphae. Our data showed ∼50-nm thick sporulation septa ∼1 μm apart but, due to the thickness of these cells, we were unable to clearly resolve the rodlet layer and PG on the cell surface (Table 1 and Figure 2C). Following septation, aerial hyphae divide into immature spores via a mechanism described as “V snapping,” which relies on turgor pressure and structural weakening of the PG to drive cell division in milliseconds (Zhou et al., 2016). In immature spore chains, the rodlet layer remained intact as a 20-nm thick sheath (Figure 2D). At this stage, an additional, ∼10-nm thick electron dense layer was observed surrounding each spore between the spore wall and the rodlet layer (Figure 3D, purple). This feature was continuous, appeared denser than the spore wall, remained associated with the mature spore and was still present in germinating spores. Since the layer we observed was tightly associated with the surface of the spore wall and appeared different than the spore wall, we speculate that it might represent a previously uncharacterized layer which we termed the spore coat. The composition and nature of this layer would be of significant interest as it may identify a new role in spore survival.

Spore maturation is defined by extensive remodeling of the spore wall, condensation of the chromosome, deposition of spore pigments, and the entrance into dormancy (Bobek et al., 2017). Once matured, spores disperse from the spore chain presumably via mechanical forces. In our cryotomograms, the spore wall appeared slightly thicker, increasing from ∼50 nm to ∼60 nm, during maturation (Table 1 and Figures 2D,E), likely directed by the SSSC (Kleinschnitz et al., 2011). In some instances, two distinct spore wall layers could be identified - a darker inner layer (∼20-nm thick) and a lighter, outer layer (∼30-nm thick). The spore wall overall has an increased percentage of 3–4 crosslinks and decreased 3–3 crosslinks compared to the PG of aerial hyphae (van der Aart et al., 2018), however, whether there are structural differences between the inner and outer layers in the spore wall remains unknown.

There has been speculation about whether the rodlet layer functions as a spore coat. For a spore coat to be protective, it needs to completely surround the spore and remain tightly associated with it. Our cryotomograms showed that after dispersal, the rodlet layer sporadically remained associated with the sides of some spores (Figure 2E), while the novel spore coat remained intact even on spores where the rodlet layer appeared detached (Figure 3D). The rodlet layer may have additional functions following spore dispersal, including modulating surface hydrophobicity to promote interactions with the flagella of motile soil bacteria and the surfaces of arthropods and springtails (Ruddick and Williams, 1972; Becher et al., 2020; Muok et al., 2020).

### Germination

Conditions that prompt spore germination are still undefined, however, heat shock and divalent cations such as Ca^2+^ and Mg^2+^ are known to stimulate germination (Hirsch and Ensign, 1976; Eaton and Ensign, 1980). Since germination defines the transition between sporulation and vegetative growth, it allowed us to make direct comparisons between the two stages. In cells undergoing germination, the spore wall was ∼60 nm thick whereas the PG of the germ tube appeared ∼ 35 nm thick (Table 1). These measurements are comparable to those for mature spores and vegetative hyphae, respectively. The vegetative PG appeared as a continuation of the inner layer of the spore wall, and the outer layer of the spore wall and spore coat could be seen peeling back from where the germ tube emerged (Figure 2F and Supplementary Movie S4). This observation suggested that the inner and outer layers of the spore wall have different structures and roles during the sporulation and germination processes.

## Conclusion

Our study shows the complete life cycle of *Streptomyces* from vegetative growth, through sporulation and germination in unprecedented detail revealed by cryo-ET. We have identified the likely *in vivo* structures of septal junctions between vegetative cells, the polarisome directing polar growth, vegetative and sporulation septa, the native PG and spore wall morphology, as well as the ultrastructure of the spore coat and rodlet layers. Our imaging reveals that cryo-ET is a powerful tool that can be applied to the *Streptomyces* system and open future areas of research. For example, imaging of wild type and Δ*parA* or Δ*filP* mutant strains could allow for the unambiguous identification of the observed filaments in the cytoplasm of vegetative cells and clarify how these proteins promote chromosome anchoring to the tip and tip rigidity, respectively. In addition, fluorescently tagging components of the polarisome and co-localizing the signals will facilitate correlative light microscopy and cryo-ET studies to further characterize the localization and *in vivo* structure of this macromolecular assembly. Lastly, looking at mutant strains that lack predicted components of the spore coat will reveal modifications of that feature at different stages of the sporulation process.

## Data Availability Statement

The raw data supporting the conclusions of this article will be made available by the authors, without undue reservation.

## Author Contributions

DS and ET designed the experiments, performed the work, processed the cryo-ET data, and revised the manuscript. DS wrote the first draft of the manuscript. ET coordinated the project. Both authors contributed to the article and approved the submitted version.

## Conflict of Interest

The authors declare that the research was conducted in the absence of any commercial or financial relationships that could be construed as a potential conflict of interest.
