# Supplementary material for: Ultrastructure of Exospore Formation in Streptomyces Revealed by Cryo-Electron Tomography
Source: Front Microbiol. 2020 Sep 24;11:581135. doi: 10.3389/fmicb.2020.581135 (PMC7541840; doi:10.3389/fmicb.2020.581135)
Supplement: Supplementary file 1 [file Data_Sheet_1.PDF]

Supplementary Material

Movie 1. Tomogram of vegetative hyphal tip. Three dimensional reconstruction of the hyphal tip from Fig. 2A.

Movie 2. The polarisome forms a layer underneath the cell membrane at hyphal tips. Tomogram of the hyphal tip in Fig. 3A. Red arrows, polarisome. Blue arrows, examples of filaments in the cytoplasm.

Movie 3. Location of septal junctions in vegetative septum. Tomogram of the vegetative septum in Fig. 3B showing the localization of the putative septal junctions (red arrows).

Movie 4. The vegetative cell wall is a continuation of the inner layers of the spore wall. Tomogram of the spore shown in in Fig. 2F.

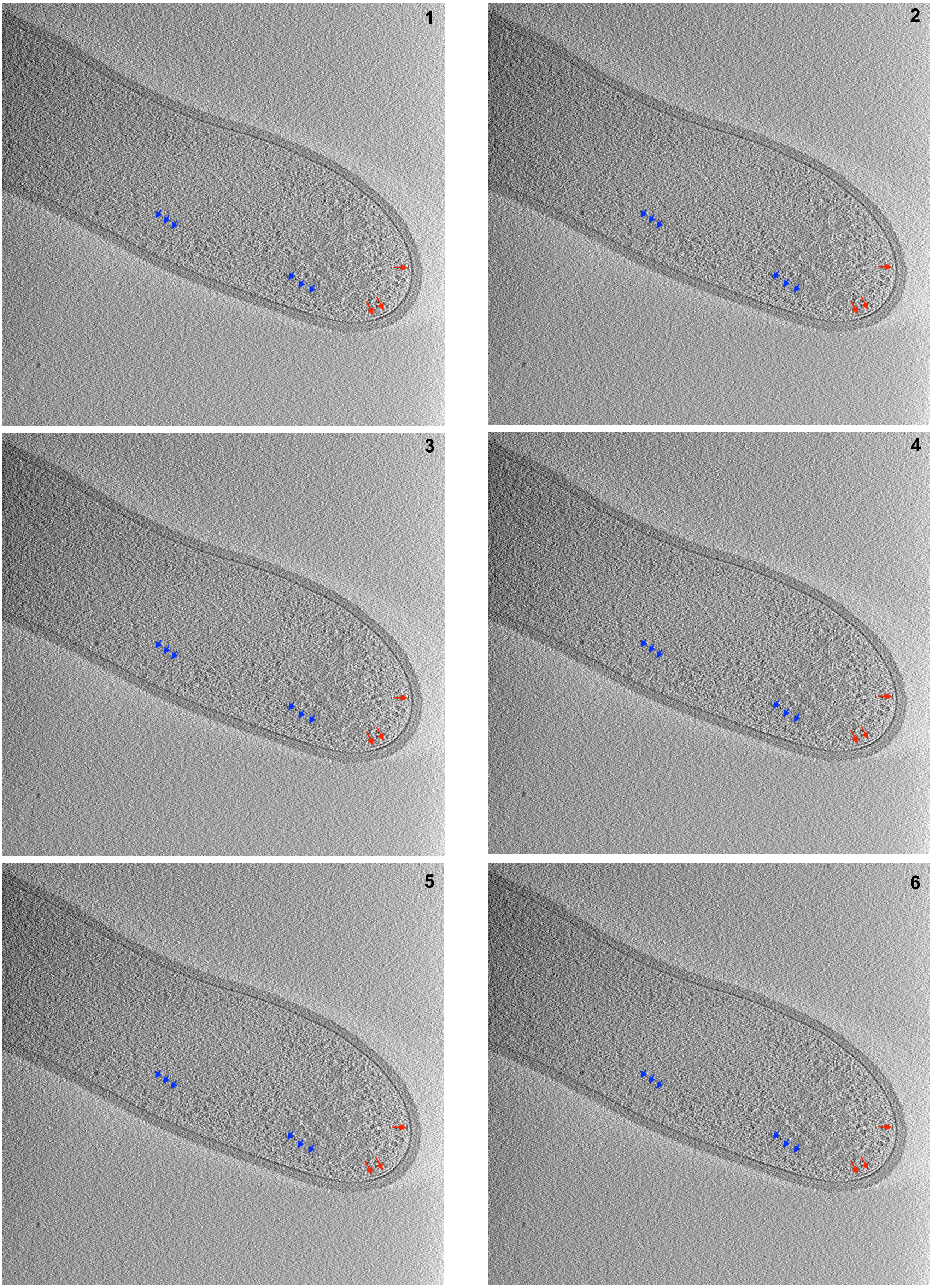

Figure S1. Additional views of the polarisome in vegetative hyphae. Consecutive slices (1-6) through the tomogram shown in Movie 2 highlighting the polarisome at hyphal tips (red arrows) and examples of filaments in the cytoplasm (blue arrows).

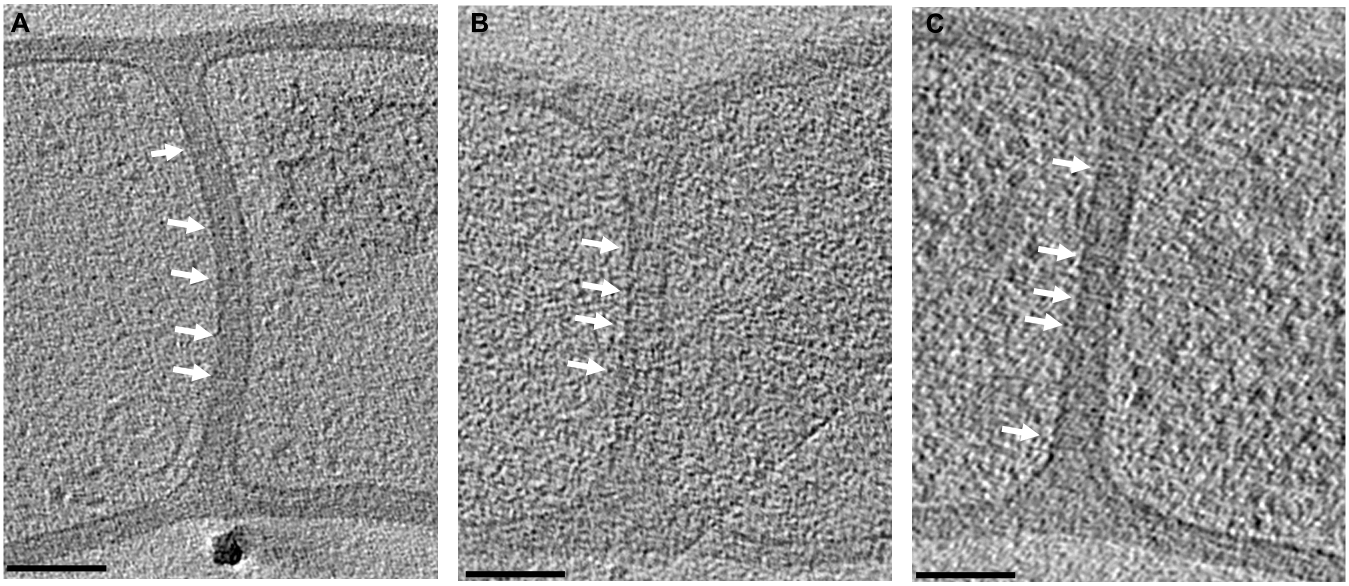

Figure S2. Additional examples of septal junctions in vegetative septa. Septal junctions (white arrows) were present in three additional vegetative septa (A-C) we examined with cryo-ET. Tomographic slices are 20 nm thick. Scale bar, 50 nm.

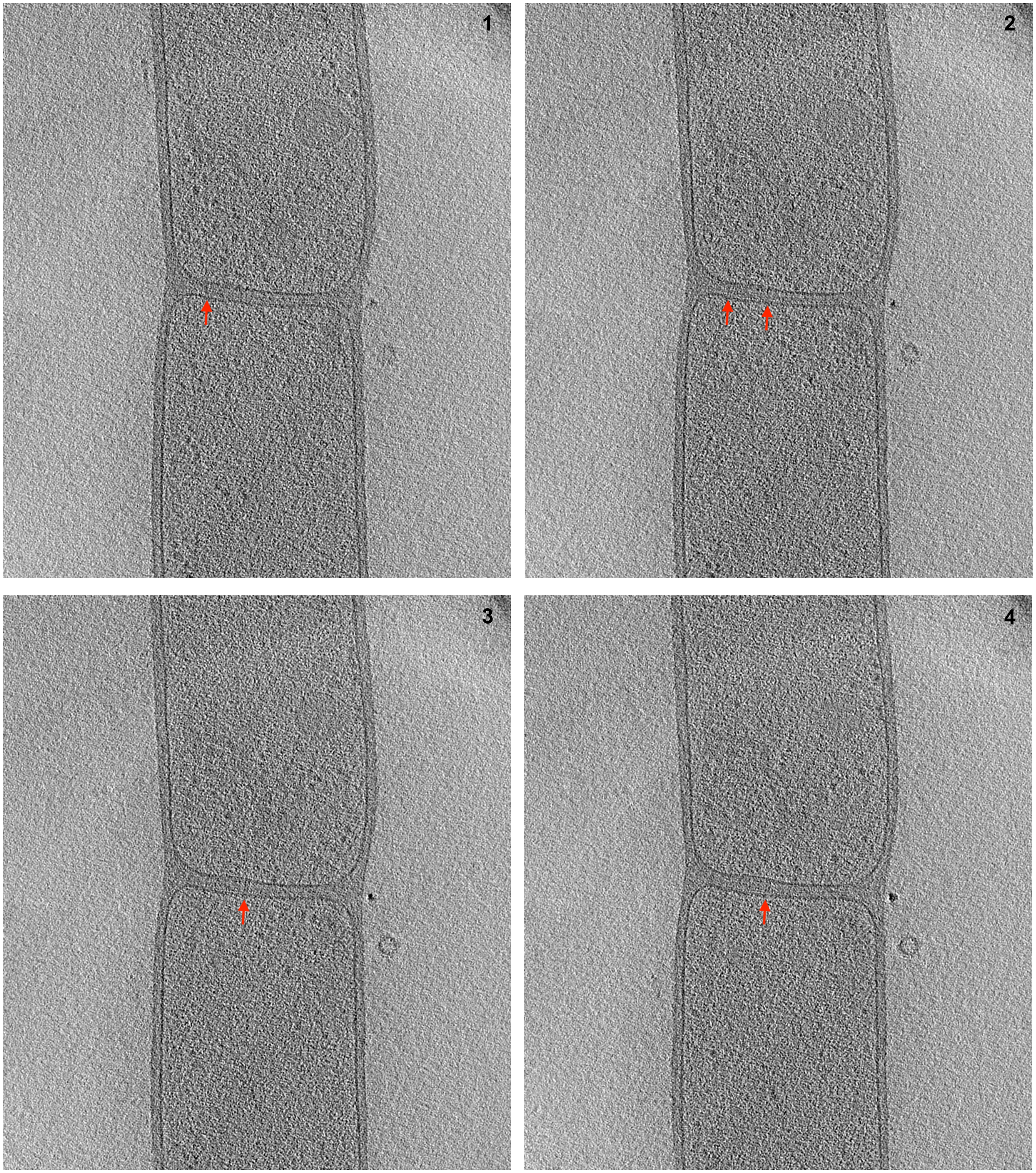

Figure S3. Additional views of septal junctions. Consecutive slices (1-4) through the tomogram shown in Movie 3 highlighting septal junctions (red arrows) between compartments of a vegetative hypha.
